# Construction and immune effect of an HPV16/18/58 trivalent therapeutic adenovirus vector vaccine

**DOI:** 10.1186/s13027-022-00417-3

**Published:** 2022-02-23

**Authors:** Bing Wan, Lu Qin, Weihong Ma, He Wang

**Affiliations:** Gynecologist Tumor Department, Guangxi Medical University Cancer Hospital, 71 Hedi Road, Zhong Shan Street, Nanning, 530021 Guangxi Zhuang Autonomous Region China

**Keywords:** Human papillomavirus type 16/18/58, E6 and E7 gene, Trivalent therapeutic vaccine, Adenovirus vaccine, Immunogenicity

## Abstract

**Objective:**

This study aims to prepare candidate vaccines for cervical cancer immunotherapy by inserting the fused genes of human papillomavirus (HPV)16/18/58 mE6E7 lacking transforming activity into an adenovirus vector and to verify its efficiency in model mice with tumor expressing the associated HPV genes.

**Methods:**

The E6/E7 genes of HPV16/18/58 were point-mutated to abolish their transforming activity, and adenovirus (AD)-HPV16/18/58 mE6E7 adenovirus vaccine was constructed. The immune effect of the adenovirus vaccine against HPV16/18/58-type tumors was analyzed by tumor morphology, enzyme linked immunosorbent assay, enzyme-linked immunospot and specific cytotoxic T lymphocyte (CTL) and T lymphocyte subsets.

**Results:**

The HPV16/18/58 mE6E7 plasmid containing point mutations was verified by quantitative real-time polymerase chain reaction (qRT-PCR), enzyme digestion and electrophoresis, and gene sequencing. qRT-PCR and Western blots verified that AD-HPV16/18/58 mE6E7 could express the HPV16 mE6E7, HPV18 mE6E7 and HPV58 mE6E7 fusion genes and proteins in cells. The results of animal experiments were as follows: In the vaccine group, the tumors formed later, the incubation period was longer, the growth was slower, growth was inhibited, and the survival period was significantly prolonged. The immunological results all showed that the vaccine could induce effective humoral and cellular immunity in mice with three types of tumors, compared with the phosphate buffered saline (PBS) group and the adenovirus-negative control (AD-NC) group, the differences were statistically significant (*P* < 0.05).

**Conclusion:**

We successfully constructed the HPV16/18/58 trivalent therapeutic adenovirus vaccine AD-HPV16/18/58 mE6E7. The AD-HPV16/18/58 mE6E7 adenovirus vaccine can protect immunized mice to a certain extent from TC-1, U14/LV-HPV18 E6E7 and U14/LV-HPV58 E6E7 cells, which contain HPV16, 18 and 58 E6 and/or E7 genes, respectively.

## Background

Cervical cancer is one of the most common malignant tumors of the female reproductive system and seriously endangers women's life and health [1]. Human papillomavirus (HPV) DNA is present in more than 99.7% of cervical cancers. HPV infection is the primary and initiating factor of the occurrence of cervical cancer [2]. Persistent infection of high-risk HPV is the most important risk factor for cervical precancerous lesions and cervical cancer [3]. Since almost all cervical cancer cells express HPV DNA and virus transforming protein, HPV protein can stimulate the body as an antigen to produce an immune response against HPV [4], suggesting that cervical cancer may be prevented and treated through vaccine immunotherapy. According to their selected antigen fragments and effects, HPV vaccines which have been put on the market or are being studied can be divided into preventive vaccines and therapeutic vaccines. Among them, the preventive vaccine takes HPV capsid protein L1 or L1/L2 chimerism as the target antigen, and the virus like particle (VLP) vaccine is commonly used at present, which mainly induces humoral immunity and is used in healthy people who are not infected with HPV. While the therapeutic vaccine takes HPV oncoprotein E6 or E7 (after eliminating the transformation activity) as the target antigen, which mainly induces cellular immunity and is used in patients who are infected with HPV or have pathological changes. HPV types 16 and 18 are considered to be the most common high-risk HPVs that cause cervical cancer. Among all types detected worldwide, HPV16 accounts for 50% and HPV18 accounts for 14% [5]. In southern China, in addition to HPV16 and 18-induced cervical cancer, HPV58 is also the dominant HPV type in Chinese women [6]. Due to the genetic differences between the various types of HPV, there is no or weak cross-protection between the various types of HPV vaccines [7], and the vaccines currently on the market are all preventive vaccines, which are not effective for cervical lesions or even cervical cancer patients who have been infected with HPV. It is gratifying that some studies have found that some therapeutic cervical cancer vaccines can effectively treat CIN II/III and cervical cancer related to HPV 16/18/58, show strong antitumor activity, provide non-surgical treatment options for CIN II/III and a new potential treatment option for cervical cancer patients [8–10]. Thus, there is an urgent demand for the development of a composite cervical cancer therapeutic vaccine including the HPV16/18/58 type to benefit Chinese patients with squamous intraepithelial lesion and cervical cancer patients suffering from high-risk HPV.

## Materials and methods

### Cell lines and animals

The TC-1 cell line was constructed by Professor Tzyy-choou Wu, Johns Hopkins School of medicine, and is a C57BL/6(H-2b) mouse lung epithelial cell cotransformed with the *E6*, *E7* and *ras* genes of HPV16 that can stably express the HPV16 E6 and E7 proteins [11], and it was cultured in 1640 medium containing 10% fetal bovine serum (FBS) and 100 U/ml penicillin and 100 g/ml streptomycin, G418 (0.4 mg/ml) and hygromycin (0.2 mg/ml) were used to maintain the expression of E6E7 plasmid. Human embryonic kidney 293A cells (Cell Bank of the Chinese Academy of Sciences, Beijing, China) and mouse cervical cancer U14 cells (derived from C57BL/6 mice) and HPV (−) (Cell Bank of the Chinese Academy of Sciences, Beijing, China) were cultured in Dulbecco’s modified Eagle’s medium (DMEM) supplemented with 10% FBS, 100 U/ml penicillin and 100 g/ml streptomycin at 37 °C with 5% CO_2_. In addition, due to the lack of commercial cell lines that can stably express E6 and E7 proteins of HPV18 and 58, our team transfected HPV18E6E7 fusion gene and HPV58E6E7 fusion gene into U14 cells through lentivirus, screened the cells that can stably express HPV18E6E7 fusion gene and HPV58E6E7 fusion gene, and named them U14/LV-HPV18E6E7 cells and U14/LV-HPV58E6E7 cells. The reagents used for cell culture were all from Thermo FisherScientific Co., Ltd., Waltham, USA. C57BL/6 female mice, 6–8 weeks old, were purchased from Hunan Slack Experimental Animal Co., Ltd., Changsha, China.

### Point mutations and plasmids

In order to lose transformation activity of E6 and E7 proteins, we genetically modified them by point mutation. HPV16E6E7 gene was modified according to Xie et al. [12]. HPV18E6E7 gene was modified according to Li et al. [13]. HPV58E6E7 gene was modified according to Wang et al. [14]. Mutations at these positions have been used in vaccine studies [12–14], and the research results confirmed that the mutant E6 protein lost the ability to degrade the P53 gene, and the mutant E7 protein lost binding activity to the pRB gene. Finally, the E6 and E7 proteins lost their transforming activity and retained their immunogenicity.

The HPV16, 18 and 58 mE6E7 fusion gene fragments after point mutation were connected to form HPV16/18/58mE6E7 fusion gene, and added EcorI restriction sites at both ends to generate and sequence the pGH-HPV16/18/58 mE6E7 plasmid. This part was completed by Beijing Kinco Biotechnology Co., Ltd., Beijing, China. All PCR primer sequences are shown in Table 1 and were synthesized by TaKaRa Bio Inc, Kusatsu, Japan.

**Table 1:** Sequences of the PCR primers in this article

Amplified target gene	Primer sequence	Amplified target gene	Primer sequence
HPV16 type mE6E7 primer	F: 5'-ATGCACCAAAAGAGAACT-3'	HPV18 type E6E7 primer	5'-ATGGCGCGCTTTGAGGATCC-3'
	R: 5'-CCATCTGTTCTCAGAAACCA-3'		5'-TGGTGTGCATCCCAGCAG-3'
HPV18 type mE6E7 primer	F: 5'-ATGGCGCGCTTTGAGGATCC-3'	HPV58 type E6E7 primer	F: 5'-GTTCCAGGACGCAGAGGAG-3'
	R: 5'-TGGTGTGCATCCCAGCAG-3'		R: 5'-TTGCTGTGCACAGCTAGGTCA-3'
HPV58 type mE6E7 primer	F: 5'-ATGTTCCAGGACGCAGAGGA-3'	Mouse β-actin primer	5'-GAGACCTTCAACACCCCAGC-3'
	R: 5'-GCCCTAGCTGTGCACAGCAA-3'		5'-CCACAGGATTCCATACCCAA-3'
HPV16 type E6 primer	F: 5'-AATGTGTGTACTGCAAGCAAC-3'	Mouse GAPDH primer	5'-AACTTTGGCATTGTGGAAGG -3'
	R: 5'-GACACAGTGGCTTTTGACAGT-3'		5'-ACACATTGGGGGTAGGAACA-3'
HPV16 type E7 primer	F: 5'-ATGGAGATACACCTACATTGC-3'		
	R: 5'-CACAACCGAAGCGTAGAGTCA-3'		

### Construction and eukaryotic expression of the AD-HPV16/18/58 mE6E7 adenovirus vaccine

#### Construction of the recombinant adenovirus shuttle plasmid ADV4-HPV16/18/58 mE6E7

We prepared the shuttle plasmid and skeleton plasmid of adenovirus particles containing the target gene fragments and cotransfected 293A cells with the transfection reagent RNAi-Mate for automatic recombination and amplification in the cells. The shuttle plasmid ADV4 vector was digested with PacI/PmeI enzymes to linearize it. We exchanged the HPV16/18/58 mE6E7 gene into an ADV4 linearized expression vector in the exchange enzyme buffer system, transformed the recombinant DNA molecule into *Escherichia coli* DH-5α competent cells, picked positive clones, and extracted plasmid DNA using a high-purity plasmid extraction kit. After the HPV16/18/58 mE6E7 fusion gene was successfully connected to the vector, we used the recombinant plasmid ADV4-HPV16/18/58 mE6E7 as a template, used the PacI/PmeI enzyme for double enzyme digestion and verified the gene sequencing.

#### Packaging and titer determination of the recombinant adenovirus particles ADV-HPV16/18/58 mE6E7

The recombinant plasmid ADV4-HPV16/18/58 mE6E7 and the adenovirus backbone plasmid pacAD59.2-100 were cotransfected into 293A cells. The cells showed complete cytopathic effect (CPE), then the cells and supernatant were collected. The 293A cells were infected again with the virus solution, the virus was amplified in large quantities, and the virus was purified using CsCl density gradient centrifugation and dialysis. The same method was used to package, amplify and purify the empty vector virus AD-NC. The viruses were saved after the viral titer was tested. The construction of the adenovirus recombinant shuttle vector, sequence determination, virus packaging and virus titer detection were completed by Suzhou GenePharma Biotech Co., Ltd., Suzhou, China.

#### Eukaryotic expression and identification of recombinant virus

The recombinant adenoviral particles AD-HPV16/18/58 mE6E7 with the HPV16/18/58 mE6E7 fusion gene and the empty vector viral particles AD-NC were infected into 293A cells at an multiple of infection (MOI) of 50, and 2.5 × 10^7^ plaque forming unit (PFU) of AD-HPV16/18/58 mE6E7 and AD-NC virus was added to each corresponding well. A blank cell control group without virus was established. The total RNA of three groups of cells was extracted by the Trizol method. cDNA synthesis and qRT-PCR were performed according to the operating instructions of the Thermo Fisher Reverse Transcription Kit (Thermo FisherScientific Co., Ltd., Waltham, USA). Ten microliters of the final reaction product was used for 2% agarose gel electrophoresis to analyze the PCR product. Then, we separately extracted the total protein of the three groups of cells and used western blotting to further identify the expression of the recombinant virus target protein. Mouse anti-HPV type 16 and 18 E6 and E7 protein monoclonal antibodies were purchased from Abcam Company, Cambridge, USA. Rabbit anti-HPV58 E6 protein polyclonal primary antibody, rabbit anti-β-actin polyclonal primary antibody, rabbit anti-GAPDH (glyceraldehyde-3-phosphate dehydrogenase) polyclonal primary antibody, goat anti-rabbit secondary antibody, and goat anti-mouse secondary antibody were purchased from Beijing Boaosen Biotech Co., Ltd., Beijing, China.

### Detection of cell lines stably expressing the HPV16/18/58 E6 and E7 genes

The transcription and translation functions of the HPV16 E6 and HPV16 E7 genes of the cells were detected by qRT-PCR and Western blot. In previous work [15], we found that HPV58E6E7 fusion gene can be stably expressed after transfection of HPV58E6E7 fusion gene into U14 cells, and it can still be expressed after subcutaneous tumor formation in C57BL/6 mice. U14 cells were transfected with virus particles containing HPV18E6E7 and HPV58E6E7, respectively. Seventy-two hours after transfection, the transfection efficiency was observed under a fluorescence microscope, and the expression of the HPV18 E6E7 fusion gene and the HPV58 E6E7 fusion gene was detected by qRT-PCR and western blots. The successfully transfected cells were named U14/LV-HPV18 E6E7 cells and U14/LV-HPV58 E6E7 cells, respectively.

### Establishment of HPV16, 18 and 58 tumor-bearing mouse models

1 × 10^7^ cells of the TC-1, U14, U14/LV-NC, U14/LV-HPV18 E6E7 and U14/LV-HPV58 E6E7 cell lines were subcutaneously inoculated on the right back of C57BL/6 female mice in 100 μl. The subcutaneous tumor formation of the mice was observed. The mice were killed 28 days after inoculation with tumor cells, and the tumor tissues were surgically stripped and stored in liquid nitrogen. The mRNA and protein expression levels of the HPV16 E6, HPV16 E7, HPV18 E6E7, and HPV58 E6E7 fusion genes in the transplanted tumor mice of each group were detected by qRT-PCR and Western blots.

### Research on the immune effect of the AD-HPV16/18/58 mE6E7 adenovirus vaccine against HPV type 16, 18 and 58-derived tumors

#### Immunization of mice

C57BL/6 female mice aged 6 ~ 8 weeks were randomly divided into 3 groups, with 45 mice/group; 15 mice were observed for tumor morphology, 15 mice were observed for survival, and 15 mice were subjected to humoral and cellular immunity tests. The immune substances of the AD-HPV16/18/58 mE6E7 vaccine group, AD-NC empty group and blank group were AD-HPV16/18/58 mE6E7 adenovirus vaccine, AD-NC empty adenovirus and sterilized PBS solution, respectively. The specific immune procedure of the treatment model is shown in Appendix Fig. 6A. Sham-treated mice were intramuscularly injected with 100 μL of PBS. The remaining two groups of mice were immunized by intramuscular injection of 10^9^ PFU (100 μL) of purified AD-NC or AD-HPV16/18/58 mE6E7 virus. All of the mice were intramuscularly immunized with the same dose three times at 7-day intervals.

#### Tumor challenge and in vivo tumor growth

C57BL/6 mice were subcutaneously injected on the backs with 1 × 10^7^ TC-1, U14/LV-HPV18 E6E7 and U14/LV-HPV58 E6E7 cells independently. The AD-HPV16/18/58 mE6E7 adenovirus vaccine was subsequently administered by tattooing on days 1, 8, and 15 after tumor challenge. Tumor growth was monitored twice each week using caliper measurements in two dimensions. The volume and growth inhibition rate of the tumors were calculated as follows: volume (mm^3^) = (width^2^ × length)/2, inhibition rate (%) = (average tumor weight of the control group-average tumor weight of the immune group)/average tumor weight of the control group × 100%. The mice were sacrificed when the tumor diameter reached 15 mm or when the tumor volume exceeded 1000 mm.

#### Immunological detection of the AD-HPV16/18/58 mE6E7 adenovirus vaccine

1. Enzyme-linked immunosorbent assay (ELISA) test: On the 7th and 14th days after the last immunization, serum from each group of mice was taken for specific antibody detection. HPV16 E7, HPV18 E7 and HPV58 E6 epitope peptides were used to pack the plates (Dakewe Co., Ltd., Shenzhen, China), The titers of specific antibodies in the sera of HPV16, 18 and 58 mice treated with vaccine, AD-NC and PBS were detected in the corresponding epitope peptide labeling wells.
  A test sample with optical density (OD) > 1 was positive, and the maximum antibody dilution showing a positive result was the serum antibody titer of the immunized mice.
2. Enzyme-linked immunospot (ELISPOT) detection: The human leukocyte antigen (HLA)-A2 restricted CTL epitope peptides from the E7 protein of HPV type 16 and 18 were synthesized and identified by Shanghai Xinhao Biotechnology Co., Ltd., Shanghai, China. HPV type 58’s E6 epitope peptides were predicted, screened, identified and stored in our research group's previous experiments. The polypeptide sequence is as follows: HPV16 E7_49-57_: RAHYNIVTF [16], HPV18 E7_7-15_: TLQDIVLHL [17], HPV58 E6_95-103_: CLNEILIRC [18]. On the 14th day after the last immunization, the spleen lymphocytes of each group of mice were separated with a lymphocyte separator (Dakewe Co., Ltd., Shenzhen, China), and the cells were seeded in 96-well plates (Dakewe Co., Ltd., Shenzhen, China) at a density of 1 × 10^6^ cells/well., and used a final concentration of 10 μg/well HPV16 E7, HPV18 E7 and HPV58 E6 epitope peptides as stimulants to stimulate each group of lymphocytes, and the level of IFN-γ secreted by the stimulated lymphocytes was detected by specific antibodies.
3. Determination of specific CTLs: On the 14th day after the last immunization, the spleen lymphocytes of each group of mice were extracted, and RPMI 1640 medium containing 10% FBS, IL-2, positive stimulus phorbol-12-myristate-13-acetate (PMA), HPV16 E7, HPV18 E7 and HPV58 E6 epitope peptide were cultured at 37 °C with 5% CO_2_ for 7 days as effector cells. Correspondingly, TC-1, U14-HPV18 E6E7, and U14-HPV58 E6E7 cells in logarithmic growth were used as target cells, and the target cell concentration was adjusted to 2 × 10^5^ cells/ml. We used a 96-well plate, added 1 × l0^4^ target cells/50 μl to each well, and then added different numbers and volumes of effector cells to form different effective target ratios (10:1, 20:1, 40:1), which were performed according to the manual operations of the CytoTox96 Non-Radioactive Cytotoxicity Assay (Promega Co., Ltd., Beijing, China). The lactate dehydrogenase (LDH) release method was used to detect specific CTLs in immunized mice. The cytotoxic killing rate (CTL%) of each group was analyzed statistically:
  Calculation formula: %CTL = (Experimental group − Effector cells-target cells)/Maximum release of target cells-spontaneous release of target cells × 100%
4. Detection of T lymphocyte subsets: On the 14th day after the last immunization, the spleen lymphocytes of each group of mice were extracted, and PBS was used to dilute the lymphocytes to 1 × 10^7^ cells/ml. We added 100 μl of cell suspension, 20 μl of Mouse T Lymphocyte Subset Antibody Cocktail and 20 μl of Isotype Control (Becton Dickinson Biotech Co., Ltd., USA) in sequence. After reaction for 30 min in the dark, we added the appropriate PBS solution to the reaction solution to shake and mix, centrifuged the sample at 800 × *g* for 10 min to discard the supernatant, added 1 ml of PBS to the precipitate again to shake and mix the cells, and detected lymphocyte subsets of each group on the CytoFLEX (Beckman Coulter Inc., USA). The results were analyzed using FlowJo X software.

### Statistics and analysis

Data are expressed as the mean ± standard deviation (s.d.), and the results were analyzed by *t* test, *x*^2^ test, nonparametric test and survival analysis. Statistical analysis was performed using statistical product and service solutions (SPSS) 26.0 statistical software. For all comparisons, differences were considered significant when *P* < 0.05.

## Results

### Obtaining the full gene of the pGH/HPV16/18/58 mE6E7 plasmid after point mutation

The results of electrophoresis (Appendix Fig. 6B) showed that the size of the HPV16 mE6E7 fusion gene fragment was approximately 768 bp, the HPV18 mE6E7 fusion gene fragment was approximately 789 bp and the HPV58 mE6E7 fusion gene fragment was approximately 741 bp, indicating that the gene synthesis was successful. HPV16/18/58 mE6E7 gene fragment (3480 bp) and pGH (3229 bp) are the same size as the target fragment (Appendix Fig. 6C). The sequencing results (Appendix Fig. 6D) confirmed that the vector was consistent with the modified target gene sequence.

### Verification of the recombinant adenovirus shuttle plasmid ADV4-HPV16/18/58 mE6E7

The electrophoresis results showed that the size of the HPV16/18/58 mE6E7 fragment was approximately 3480 bp, indicating that the target sequence was successfully linked to the ADV4 vector (Appendix Fig. 6E). The digested HPV16/18/58 mE6E7 fragments were purified and collected for sequencing. The sequencing peak spectrum was consistent with the target gene (Appendix Fig. 6F), indicating that the recombinant plasmid was successfully generated.

### Packaging and titer determination results of the recombinant adenovirus particles ADV4-HPV16/18/58 mE6E7

**Fig. 1:**
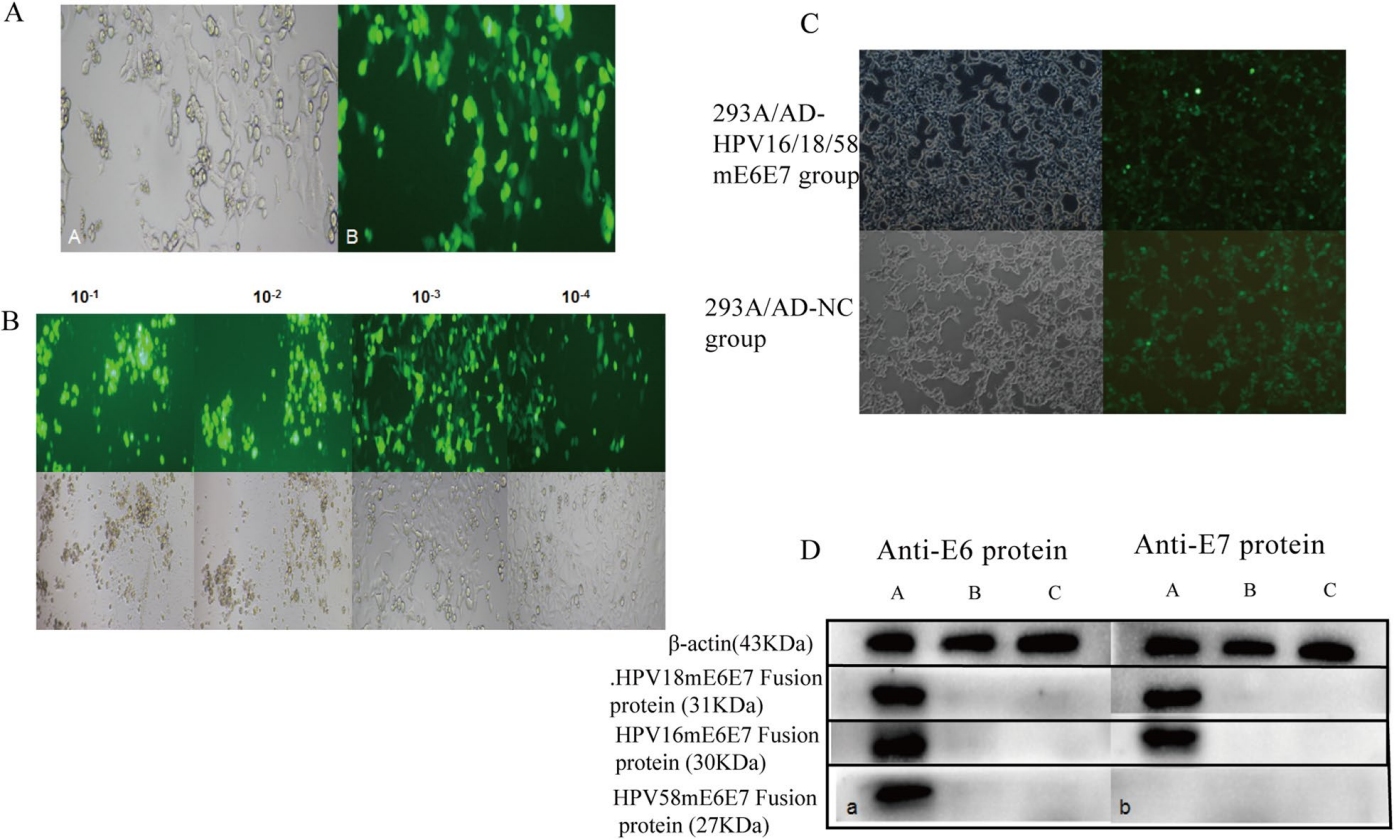
Construction of the recombinant adenovirus ADV4-HPV16/18/58 mE6E7. **A** The recombinant adenovirus shuttle plasmid ADV4-HPV16/18/58 mE6E7 and the adenovirus backbone plasmid were cotransfected into 293A cells. **B** CPE phenomenon after 293A cells were infected with different dilutions of viruses. **C** AD-HPV16/18/58 mE6E7 and AD-NC viruses were transfected into 293A cells. **D** The protein expression of E6 and E7 in the 293A/AD-HPV16/18/58 mE6E7 cells was detected by western blots. a: anti-E6 protein b: anti-E7 protein, A: 293A/AD-HPV16/18/58 mE6E7 group, B: 293A/AD-NC group, C: 293A group

After 7 days, a high level of green fluorescence was observed in the cells, indicating that the recombinant adenovirus ADV4-HPV16/18/58 mE6E7 and the empty vector virus AD-NC were successfully packaged (Fig. 1A). The transfected 293A cells showed complete CPE that the cells became round and some cells floated (Fig. 1B). After 36–48 h of infection with adenovirus, the titer of the amplified adenovirus was calculated according to the formula and was approximately 1 × 10^9^ PFU/ml. 293A cells were infected with AD-HPV16/18/58 mE6E7 and AD-NC virus for 36–48 h, many green fluorescent cells were observed under a fluorescence microscope, and the CPE phenomenon was visible in the cells (Fig. 1C).

The RT-PCR analyzed that the 293A/AD-HPV16/18/58 mE6E7 cell group had corresponding bands at the position of the HPV16 or 18 or 58 mE6E7 fragments, while the 293A/AD-NC cells and 293A cells had no corresponding bands. Western blot analysis showed that (Fig. 1D) the HPV16 E6, HPV18 E7, HPV18 E6 and HPV58 E6 proteins were expressed in the 293A/AD-HPV16/18/58 mE6E7 cells but not in the control 293A/AD-NC cells or 293A cells.These indicated that the HPV16, 18, and 58 mE6E7 genes were successfully expressed in the 293A/AD-HPV16/18/58 mE6E7 cells.

### The analytical results of cell lines stably expressing the HPV16/18/58 E6 and E7 genes

#### Verification of TC-1 cell line stably expressing the HPV16 E6 and HPV16 E7 genes

**Fig. 2:**
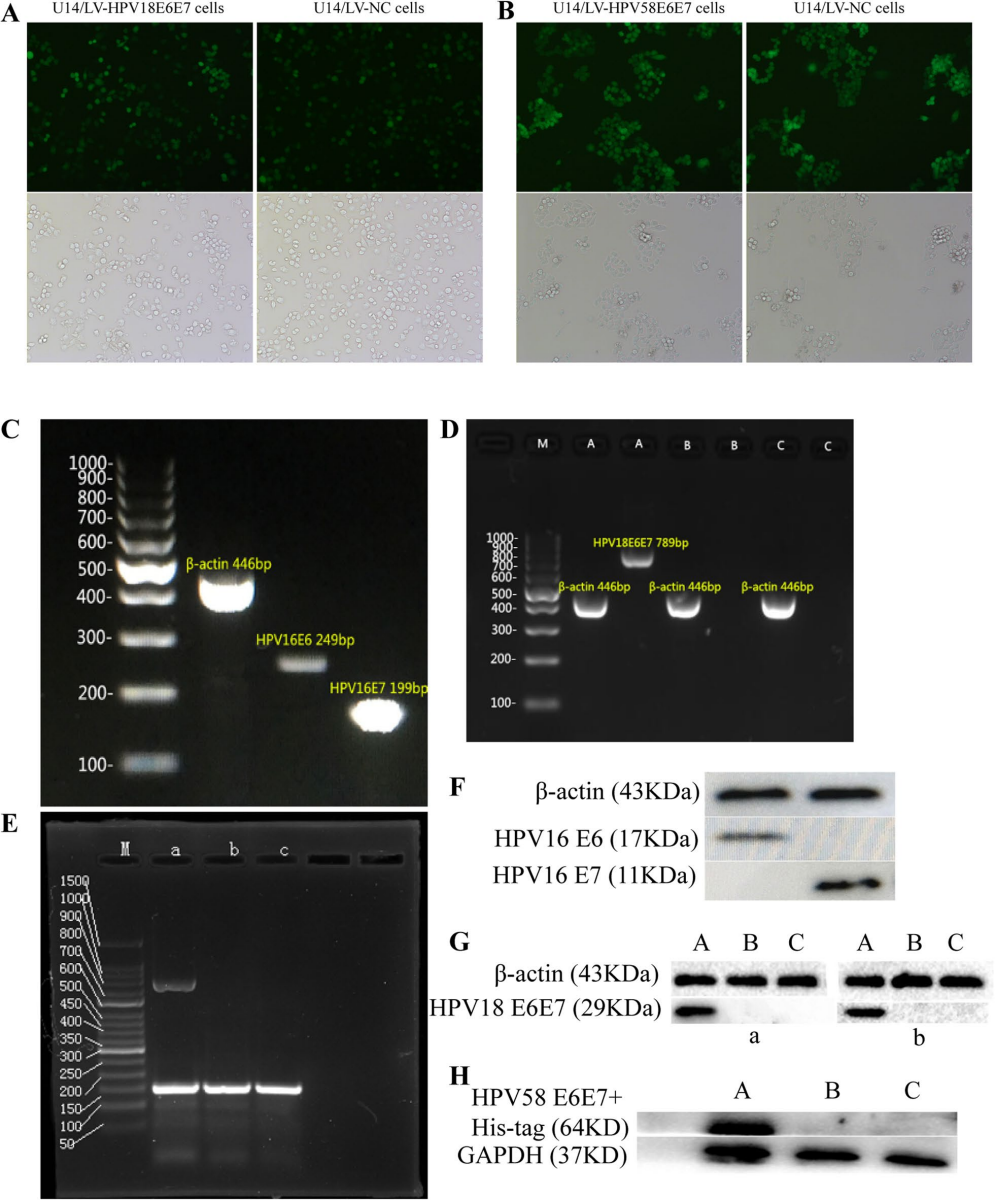
Construction of a mouse cervical cancer U14 cell line stably expressing the HPV16/18/58 E6E7 fusion protein. **A** Fluorescence microscopy was used to detect the expression of green fluorescent protein in the U14/LV-HPV18 E6E7 cells and U14/LV-NC cells. **B** Fluorescence microscopy was used to detect the expression of green fluorescent protein in the U14/LV-HPV58 E6E7 cells and the U14/LV-NC cells. **C** The expression of the HPV16 E6 and HPV16 E7 genes was detected in TC-1 cells by RT-PCR (HPV E6 249 bp, HPV E7 199 bp). **D** The expression of the HPV18 E6E7 fusion gene in each group was detected by RT-PCR. M: DNA ladder (100 bp) A: U14/LV-HPV18 E6E7 group (HPV18 E6E7 789 bp), B: U14/LV-NC group, C: U14 group. **E** The expression of the HPV58 E6E7 fusion gene in each group was detected by RT-PCR M: DNA marker (50 bp) a: U14/LV-HPV58 E6E7 group (HPV58 E6E7 741 bp), b: U14/LV-NC group, c: U14 group. **F** Western blot analysis showed that the HPV16 E6 and E7 proteins in TC-1 cells can be expressed. **G** Western blot analysis showed that the HPV18 E6E7 protein in the U14/LV-HPV18 E6E7 cells could be detected. A: U14/LV-HPV18 E6E7 group, B: U14/LV-NC group C: U14 group, a: anti-E6 protein, b: anti-E7 protein. **H** Western blot analysis confirmed that the U14/LV-HPV58 E6E7 fusion protein was expressed in cells from the A: U14/LV-HPV58 E6E7 group, B: U14/LV-NC group, and C: U14 group

The results of RT-PCR electrophoresis confirmed that both the HPV16 E6 and E7 genes were expressed in TC-1 cells. The HPV16 E6 gene fragment was approximately 249 bp, and the HPV16 E7 gene was approximately 199 bp (Fig. 2C). Western blot analysis confirmed that the HPV16 E6 and E7 proteins were expressed in TC-1 cells (Fig. 2F).

#### Construction of the U14 cell line stably expressing the HPV18/58 E6E7 fusion protein

Seventy-two hours after transfection, the cells exhibited green fluorescence under a fluorescence microscope, and the rate of virus transfection was over 90%, while U14 cells did not show green fluorescence (Fig. 2A, B). RT-PCR results showed that in the U14/LV-HPV18 E6E7 group (Fig. 2D), the HPV18 E6E7 fusion gene was observed at 789 bp, but this band was not detected in the U14/LV-NC group and U14 group. In the U14/LV-HPV58 E6E7 group (Fig. 2E), the HPV58 E6E7 fusion gene was approximately 741 bp, but it was not expressed in the U14/LV-NC cells and U14 cells. The results of western blotting (Fig. 2G) confirmed that the HPV18 E6E7 fusion protein (29 kDa) was expressed in the U14/LV-HPV18 E6E7 cells, while the expression of the HPV18 E6E7 protein was not observed in the U14/LV-NC cells and U14 cells. The HPV58 E6E7 + His-tagged fusion protein bands showed that the HPV58 E6E7 fusion protein was 27 kDa and the His-tagged protein was 37 kDa in U14/LV-HPV58 E6E7 cells, but there was no such band in the U14/LV-NC cells and U14 cells (Fig. 2H).

### Establishment of an animal model

**Fig. 3:**
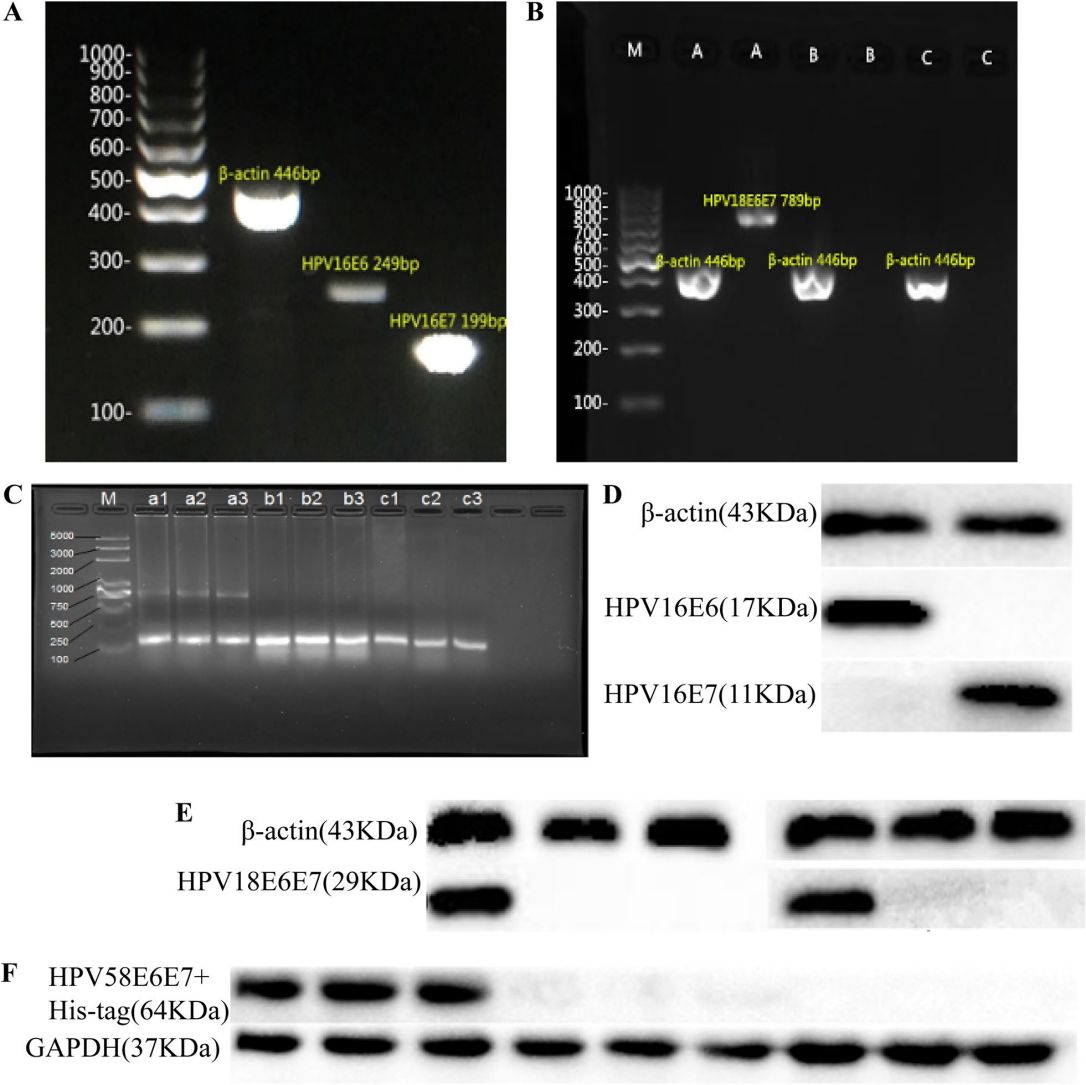
Establishment of a mouse tumorigenesis model and detection of related gene expression. **A** The HPV16 E6 and HPV16 E7 genes in the tissues of the TC-1 transplantation tumor group were detected by RT-PCR (HPV16 E6 249 bp, HPV16 E7 199 bp). **B** HPV18 E6E7 fusion gene expression in the tumor tissues of the three groups was detected by RT-PCR. M: DNA ladder (100 bp) A: U14/LV-HPV18 E6E7 group (HPV18 E6E7 789 bp), B: U14/LV-NC group, C: U14 group. **C** HPV58 E6E7 fusion gene expression in the tumor tissues of the three groups was detected by RT-PCR. M: DNA marker a1, a2, a3: U14/LV-HPV58 E6E7 group (HPV58 E6E7 741 bp), b1, b2, b3: U14/LV-NC group, c1, c2, C3: U14 group. **D** Western blot results of the HPV16 E6 and E7 proteins in the tissues of the TC-1 transplantation tumors. **E** Western blot results of the HPV18 E6 and HPV18 E7 proteins in three groups of mouse tumor tissues: A: U14/LV-HPV18 E6E7 group, B: U14/LV-NC group, C: U14 group. **F** Western blot results of the HPV58 E6E7 + His-tag fusion protein in three groups of tumor tissues: a1, a2, a3: U14/LV-HPV58 E6E7 group, b1, b2, b3: U14/LV-NC group, c1, c2 c3: U14 group

The tumor was palpated at the inoculation site 7 to 9 days after cell inoculation, and the tumor formation rate of the transplanted tumor was 100%. 28 days after tumor cells inoculation, the mice were killed and the tumor tissues were surgically stripped for RT-PCR and Western-blot experiment. The results of RT-PCR showed that in the TC-1 group, HPV16 E6 gene band fragments were approximately 249 bp, and HPV16 E7 gene band fragments were approximately 199 bp (Fig. 3A). In the U14/LV-HPV18 E6E7 group, the HPV18 E6E7 fusion fragment was 789 bp, but it was not detected in the U14/LV-NC group and U14 group (Fig. 3B). In the U14/LV-HPV58 E6E7 group, the HPV58 E67 fusion fragment was approximately 741 bp in size, while the U14/LV-NC group and U14 group did not have this band (Fig. 3C). Western blot analysis confirmed that the HPV16 E6 (17 kDa) and HPV16 E7 (11 kDa) proteins were expressed in the tumor tissues of the TC-1 cell xenograft group (Fig. 3D). The HPV18 E6 protein (17 kDa) and the HPV18 E7 protein (12 kDa) were expressed in the tumor tissues of the mice in the U14/LV-HPV18 E6E7 group but not in the U14/LV-NC and U14 groups (Fig. 3E). In the mice tumor tissue of the U14/LV-HPV58 E6E7 group, there were proteins with a molecular weight of approximately 64 kDa (the theoretical molecular weight of the HPV58 E6E7 + His tag target fusion protein) that could specifically bind to the anti-HPV58 E6 antibody, while there was no such band in the control group of U14/LV-NC and U14 cells (Fig. 3F).

### The results of the AD-HPV16/18/58 mE6E7 adenovirus vaccine administration for antitumor protection

**Fig. 4:**
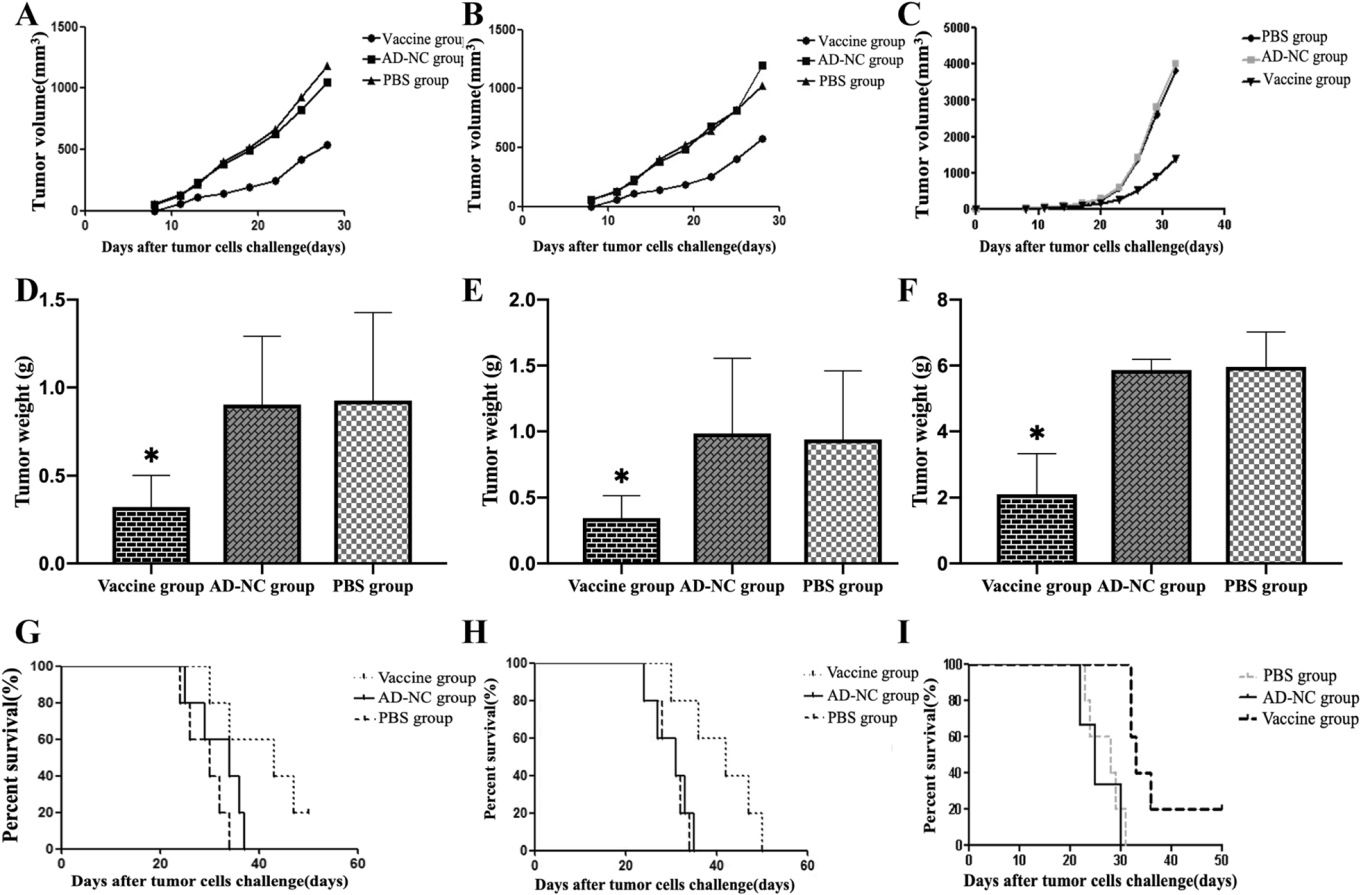
Tumor growth and survival time of the HPV16, 18 and 58 tumor-bearing mice. **A** Tumor volume in each group of mice in the HPV16 (TC-1 cells) group. Tumor volume in vaccine group, AD-NC group and PBS group were significant (*P* < 0.05). **B** Tumor volume in each group of mice in the HPV18 (U14/LV-HPV18 E6E7 cells) group. Tumor volume in vaccine group, AD-NC group and PBS group were significant (*P* < 0.05). **C** Tumor volume in each group of mice in the HPV58 (U14/LV-HPV58 E6E7 cells) group. Tumor volume in vaccine group, AD-NC group and PBS group were significant (*P* < 0.05). **D** Tumor weight in each group of mice in the HPV16 (TC-1 cells) group. Tumor weight in vaccine group (11.4 ± 0.548 g), AD-NC group (8.2 ± 0.447 g) and PBS group (8.0 ± 0.707 g) were significant (*P* < 0.05). **E** Tumor weight in each group of mice in the HPV18 (U14/LV-HPV18 E6E7 cells) group. Tumor weight in vaccine group (11.2 ± 0.447 g), AD-NC group (7.8 ± 0.447 g) and PBS group (7.6 ± 0.547 g) were significant (*P* < 0.05). **F** Tumor weight in each group of mice in the HPV58 (U14/LV-HPV58 E6E7 cells) group. Tumor weight in vaccine group (12.2 ± 0.837 g), AD-NC group (9.6 ± 0.548 g) and PBS group (9.6 ± 1.140 g) were significant (*P* < 0.05). **G** Percent survival in each group of mice in the HPV16 (TC-1 cells) group. Percent survival in vaccine group, AD-NC group and PBS group were significant (*P* < 0.05). **H** Percent survival in each group of mice in the HPV18 (U14/LV-HPV18 E6E7 cells) group. Percent survival in vaccine group, AD-NC group and PBS group were significant (*P* < 0.05). (I) Percent survival in each group of mice in the HPV58 (U14/LV-HPV58 E6E7 cells) group. Percent survival in vaccine group, AD-NC group and PBS group were significant (*P* < 0.05)

The average tumor volume and weight of the vaccinated HPV16(+), 18(+) and 58(+) tumor-bearing mice were significantly lower than those of the AD-NC and PBS groups (*P* < 0.05), while the average tumor volume and weight of the AD-NC and PBS mice were not significantly different (*P* > 0.05) (Table 2; Fig. 4A–F). Compared with PBS control group, the tumor inhibition rates of HPV16 (+), HPV18 (+) and HPV58 (+) mice in the vaccine group were 65.23%, 63.40% and 64.71%, respectively, and AN-NC group were 2.59%, 4.79%, 1.63%.The survival period of three vaccinated tumor-bearing mice was significantly longer than that of the AD-NC and PBS groups, and the difference was statistically significant (Fig. 4G–I).

**Table 2:** The average tumor weight of the HPV16, 18 and 58 groups of tumor-bearing mice after immunization with different immune substances (g)

Group	TC-1 group	U14/LV-HPV18 E6E7 group	U14/LV-HPV58 E6E7 group
AD-HPV16/18/58 mE6E7 vaccine group	0.322 ± 0.18*	0.344 ± 0.17	2.103 ± 1.220*
AD-NC group	0.902 ± 0.39	0.985 ± 0.57	5.862 ± 0.323
PBS group	0.926 ± 0.50	0.940 ± 0.52	5.959 ± 1.059

Control groups are the AD-NC group and PBS group

*Compared with that of the control group, the average tumor weight of the mice in the vaccine group was significantly reduced (*P* < 0.05)

### Mouse-specific antibody detection

**Fig. 5:**
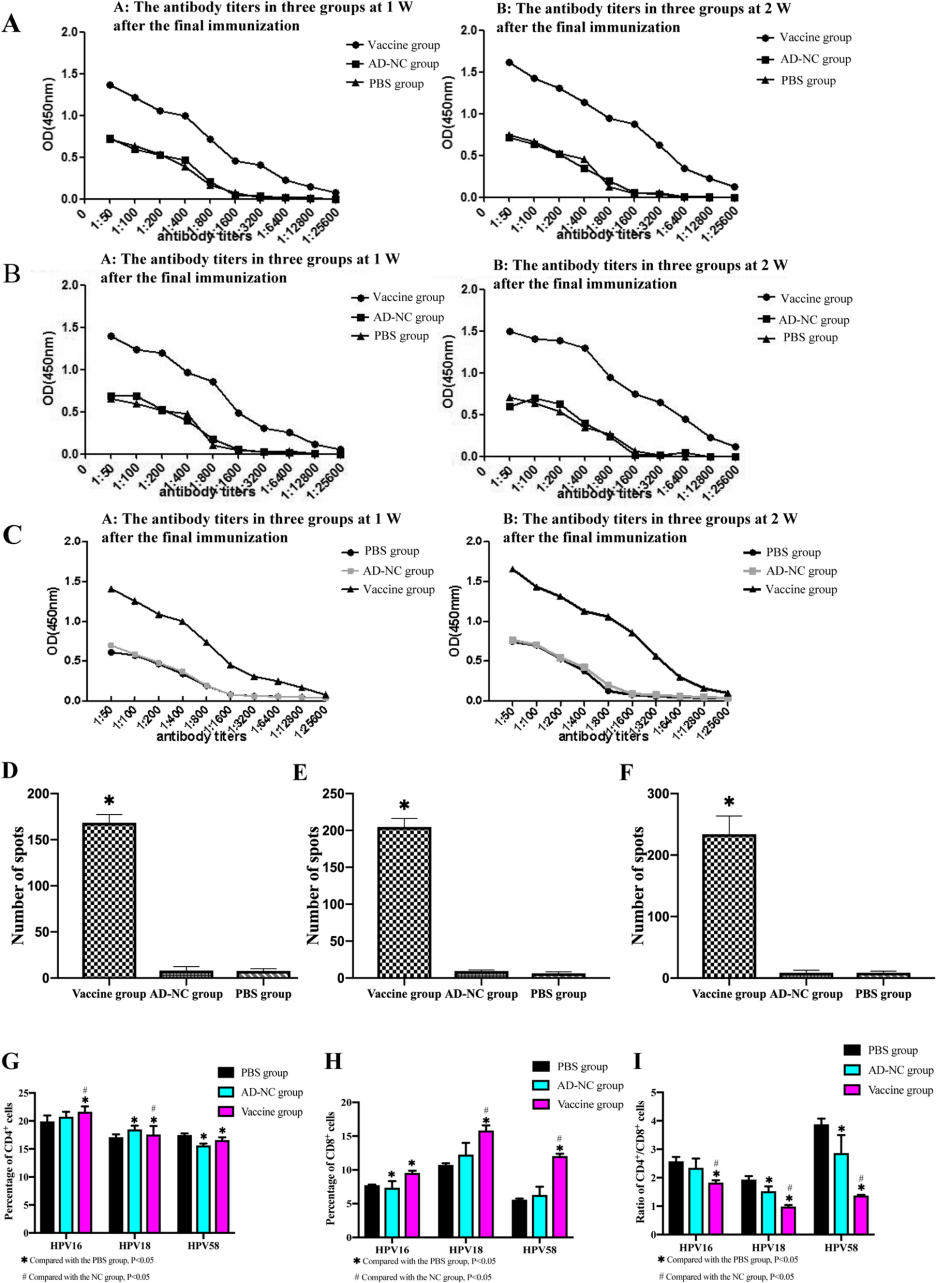
Immunological results of the AD-HPV16/18/58 mE6E7 adenovirus vaccine anti-tumor. **A** The serum antibody results of each group of mice in the HPV16 (TC-1 cell) group A: Mouse serum antibody results 1 week after the last injection, B: Mouse serum antibody results 2 weeks after the last injection. **B** The serum antibody results of each group of mice in the HPV18 (U14/LV-HPV18 E6E7 cell) group A: Mouse serum antibody results 1 week after the last injection, B: Mouse serum antibody results 2 weeks after the last injection. **C** The serum antibody results of each group of mice in the HPV58 (U14/LV-HPV58 E6E7 cell) group A: Mouse serum antibody results 1 week after the last injection, B: Mouse serum antibody results 2 weeks after the last injection. **D** The number of ELISPOT spots of IFN-γ in each group of HPV16 (TC-1 cell) mice. 168.33 ± 8.963 spots for the vaccine group, 8.00 ± 4.359 spots for the AD-NC group and 7.67 ± 2.517 spots for the PBS group were significant (*P* < 0.05). **E** The number of ELISPOT spots of IFN-γ in each group of HPV18 (U14/LV-HPV18 E6E7 cells) mice. 204.67 ± 11.676 spots for the vaccine group, 9.33 ± 1.528 spots for the AD-NC group and 6.33 ± 2.062 spots for the PBS group were significant (*P* < 0.05). **F** The number of ELISPOT spots of IFN-γ in each group of HPV58 (U14/LV-HPV58 E6E7 cells) mice. 234.00 ± 29.614 spots for the vaccine group, 8.67 ± 4.041 spots for the AD-NC group and 8.67 ± 2.517 spots for the PBS group were significant (*P* < 0.05). **G** Changes in T lymphocyte subsets in each group of mice in the HPV16 (TC-1 cell) group. **H** Changes in T lymphocyte subsets in each group of mice in the HPV18 (U14/LV-HPV18 E6E7 cells) group. **I** Changes in T lymphocyte subsets in each group of mice in the HPV58 (U14/LV-HPV58 E6E7 cell) group

On the 7th day after the last immunization, the sera of the vaccine-immunized HPV16, 18 and 58 tumor-bearing mice all showed specific antibodies with a serum titer of 1:12,800, and on the 14th day after the last immunization, the serum antibody titer was 1:25,600. The serum antibody titer of the tumor-bearing mice in each cell group immunized with PBS and AD-NC did not increase significantly (1:800) (Fig. 5A–C).

### The level of mouse IFN-γ-secreting cells was detected by ELISPOT

Vaccine-immunized HPV16, 18 and 58 groups of tumor-bearing mice were used to assess specific T lymphocytes. The number of spots formed by IFN-γ secreting cells was 168.33 ± 8.963/1 × 10^5^ lymphocytes, 204.67 ± 11.676/1 × 10^5^ lymphocytes and 234 ± 29.614/1 × 10^5^ lymphocytes, respectively, while the AD-NC empty group had 8 ± 4.359/1 × 10^5^ lymphocytes, 9.33 ± 1.528/1 × 10^5^ lymphocytes, and 8.67 ± 4.041 cells/1 × 10^5^ lymphocytes, and the PBS blank control group had 7.67 ± 2.517 cells/1 × 10^5^ lymphocytes, 6.33 ± 2.062 cells/1 × 10^5^ lymphocytes and 8.67 ± 2.517/1 × 10^5^ lymphocytes, respectively. The spot numbers of the vaccine group were significantly different from those of the AD-NC and PBS groups (*P* < 0.05) (Fig. 5D–F; Table 3). The results show that the vaccine can effectively activate the mouse body to produce specific T lymphocytes, so as to specifically kill HPV16/18/58 tumor cells.

**Table 3:** The number of ELISPOT spots of IFN-γ on splenic lymphocytes of mice in each of the three groups

Group	HPV16	HPV18	HPV58
AD-HPV16/18/58 mE6E7 vaccine group	168.33 ± 8.963*	204.67 ± 11.676*	234.00 ± 29.614*
AD-NC group	8.00 ± 4.359	9.33 ± 1.528	8.67 ± 4.041
PBS group	7.67 ± 2.517	6.33 ± 2.062	8.67 ± 2.517

*Compared with that of the blank group, the number of ELISPOT spots on the spleen lymphocytes of the mice in this group was significantly increased (*P* < 0.05)

### Specific CTL detection

Specific CTLs were detected in the three groups of vaccinated tumor-bearing mice, and the average killing rate increased with increasing effective target ratio. The average killing rates of the vaccinated HPV16, 18, and 58 mice with effective target ratios of 10:1, 20:1, and 40:1 were 11.678%, 21.201% and 30.160%; 14.682%, 22.871% and 30.890%; and 15.381%, 22.807% and 33.050%, respectively. However, the mice vaccinated with AD-NC or PBS could not stimulate specific CTLs, the vaccine group had significantly higher values than the latter two groups, and the difference was statistically significant (*P* < 0.05). There was no significant difference in the average kill rate between the latter two groups with different effect-to-target ratios (Table 4). The results showed that the vaccine activated the immune system by active immunization, which could significantly enhance the body to produce specific CTL with E6 and E7 antigens as target molecules, so as to kill HPV16/18/58 tumor cells, but had no damage to normal tissues.

**Table 4:** The results of specific CTLs of different substances and different effective target ratios in the HPV16, 18 and 58 groups of tumor-bearing mice

Effective target ratio	HPV16			HPV18			HPV58		
	Vaccine group	AD-NC group	PBS group	Vaccine group	AD-NC group	PBS group	Vaccine group	AD-NC group	PBS group
10:1	11.678%	3.857%	1.867%	14.682%	4.509%	3.031%	15.381%	3.115%	2.501%
20:1	21.201%	4.840%	6.798%	22.871%	4.509%	4.906%	22.807%	2.277%	4.105%
**40:1**	30.160%*	2.580%	3.136%	30.890%*	4.467%	5.563%	33.050%*	3.102%	6.168%

*After the three groups of tumor-bearing mice were vaccinated, the specific CTL stimulated by the vaccine group was significantly higher than that of the AD-NC or PBS group, and the difference was statistically significant (*P* < 0.05). There was no significant difference in the average killing rate between the latter two groups with different target ratios

### T lymphocyte subset detection

Cellular immunity is more important than humoral immunity in anti HPV infection. There are a large number of monocytes infiltrating in cervical cancer, mainly CD8^+^ cytotoxic T cells, which are just the main component of CTL. The results showed that the T lymphocytes in the spleens of the three groups of vaccinated tumor-bearing mice all changed significantly (Fig. 5G–I; Table 5). In the HPV16 tumor-bearing mice, compared with those of the PBS blank control group and AD-NC group, the CD4^+^/CD8^+^ ratio in the vaccine group were significantly reduced, while the ratio of CD8^+^ lymphocytes was significantly increased compared with those of the PBS blank control group (*P* < 0.05). In the HPV18 tumor-bearing mice, the CD4^+^ lymphocyte ratio and the CD4^+^/CD8^+^ ratio of the vaccine group were significantly decreased, while the CD8^+^ lymphocyte ratio was significantly increased compared with those of the PBS blank control group and the AD-NC group (*P* < 0.05). In the HPV58 tumor-bearing mice, the CD4^+^/CD8^+^ ratio of the vaccine group were significantly decreased, while the CD8^+^ lymphocyte ratio was significantly increased compared with those of the PBS blank control group and the AD-NC group (*P* < 0.05).

**Table 5:** Percentage of CD4^+^, CD8^+^ cells and ratio of CD4^+^/CD8^+^ in the HPV16, 18 and 58 groups of tumor-bearing mice

Group	HPV16			HPV18			HPV58		
	CD4^+^	CD8^+^	CD4^+^/CD8^+^	CD4^+^	CD8^+^	CD4^+^/CD8^+^	CD4^+^	CD8^+^	CD4^+^/CD8^+^
PBS group	19.920 + 1.069	7.730 + 0.098	2.578 + 0.153	20.757 + 0.886	10.747 + 0.225	1.933 + 0.122	21.660 + 0.938	5.583 + 0.170	3.881 + 0.198
AD-NC group	17.110 + 0.503	7.357 + 0.981^a^	2.354 + 0.317	18.497 + 0.657^a^	12.253 + 1.762	1.526 + 0.169^a^	17.577 + 1.509^a^	6.287 + 1.227	2.870 + 0.630^a^
Vaccine group	17.493 + 0.287^a,b^	9.560 + 0.310^a^	1.832 + 0.081^a,b^	15.633 + 0.329^a,b^	15.833 + 0.761^a,b^	0.989 + 0.052^a,b^	16.583 + 0.497^a^	12.047 + 0.345^a,b^	1.377 + 0.020^a,b^

^a^Compared with the PBS group, *P* < 0.05

^b^Compared with the NC group, *P* < 0.05

## Discussion

There are currently more than 100 subtypes of HPV, and the distribution has obvious regional differences [19]. HPV infection types vary in different countries and regions. The top five most common subtypes in China are HPV type 16, 18, 33, 52 and 58 [20], especially in southern China, such as Guangxi, where HPV type 58 has surpassed HPV type 18 as the second most common subtype [21], even equal to the detection rate of HPV type 16. In the same period, the detection rate of HPV type 58 was only 0 ~ 3% [22, 23], and there was little research on HPV type 58. At present, the research on HPV related vaccine is mainly HPV types 16 and 18, and the research on HPV type 58 vaccine is still in an immature stage. In addition, due to the differences among HPV genes, there is no cross protection or weak cross protection among HPV vaccines [24], and the vaccines currently on the market are preventive vaccines, which are ineffective for patients who have been infected with HPV or even have squamous intraepithelial lesion and cervical cancer. Therefore, we selected the three most common HPV pathogenic subtypes HPV16, 18 and 58 in China, especially in southern China, to develop a multivalent therapeutic vaccine containing HPV16/18/58, which will benefit Chinese patients with high-risk HPV associated squamous intraepithelial lesions and cervical cancer.

The HPV E6 and E7 proteins are highly expressed in a series of stages from the initial stage of infection to the occurrence of malignant tumors, and both E6 and E7 proteins can induce the body to produce strong and continuous specific CTLs targeting viral antigens [25, 26]. Therefore, E6 and E7 proteins are ideal antigen targets for HPV therapeutic vaccines, which are suitable for immunotherapy and prevention of cervical cancer caused by HPV infection. However, the prerequisite is to eliminate the transformation activity of E6 and E7 genes to eliminate the potential cancer risk [27]. The active sites of the E6 and E7 proteins involved in degradation of P53 and PRb are located in their zinc finger structure regions. Changes or deletions of certain specific amino acids in this region can weaken or eliminate the E6 and E7 protein-mediated degradation of P53 and pRb to varying degrees. In this study, based on the literatures [28–32], we adopted the following strategy for site-directed mutation. For the HPV16 E6 protein, Leu at position 57 was mutated to Gly, Cys at position 113 was mutated to Arg, and Cys at position 24 and Glu at position 26 of the HPV16 E7 protein were mutated to Gly. For the HPV18 E6 protein, the 52nd residue Leu was mutated to Gly, the 138th residue Cys was mutated to Gly, and the 27th and 98th Cys residues of the HPV18 E7 protein were mutated to Gly. For HPV58 E6 protein, Leu50 and the Cys63 and 106 were all mutated to Gly, and Glu24 and 26 and Cys92 of the HPV58 E7 protein were all mutated to Gly. Mutations at these sites have been confirmed in previous HPV therapeutic vaccine studies [9–11].The mutant E6 protein loses its ability to degrade P53, and the E7 protein loses the ability to bind to pRB. These changes result in complete loss of the transforming activity of the E6 and E7 proteins and reduce their carcinogenic potential but effectively retain their immune antigenicity; thus, they still have the ability to induce HPV-specific cell-mediated immune responses in the body. Therefore, these proteins can become the target antigen for the development of HPV16, 18, and 58 therapeutic vaccines.

For gene vaccine construction, in addition to the correct selection of the most specific, efficient, and broad-spectrum target antigens, proper expression vectors are extremely important. The current therapeutic vaccines for HPV-related cancers mainly include peptides, proteins, carriers (nontoxic, attenuated or attenuated viruses or bacteria) and nucleic acid vaccines [33]. Adenovirus vectors have high efficiency of gene transfer, and the transduction efficiency in vitro is usually close to 100%. These vectors will not integrate with the genome of the host cell; furthermore, they are expressed instantaneously and very safe [34]. These vectors can endogenously express protective antigen genes in infected cells, correct post-translational modification, compensate for the lack of spatial structure of peptide vaccines, and stimulate the body's CTL response. Therefore, ADV4, which is a replication-deficient adenovirus modified by the AD5 adenovirus with deletion of the E1 gene, was used in this experiment. This vector system has no left-side ITR, packaging signal or E1 sequence, will not produce replication-deficient adenovirus (RCA), and shows good safety. What’s more, the vector does not require homologous recombination and plaque separation in bacteria during the virus recombination process, so the operation is simple and convenient; moreover, the adenovirus expression system shows fast growth, reducing the risk of recombination to form a wild-type replicable adenovirus. Its system comprises the shuttle plasmid ADV4, the backbone plasmid pacAD59.2-100 and 293A cells. We insert the foreign target gene HPV16/18/58 mE6E7 into the shuttle plasmid ADV4, it was cotransfected with the backbone plasmid pacAD59.2-100 into 293A cells, and its eukaryotic expression in cells was verified by RT-PCR and western blot experiments. The shuttle plasmid ADV4 and the backbone plasmid pacAD59.2-100 undergo homologous recombination in the cell, thereby packaging and producing adenovirus particles. The protein shell of the recombinant adenovirus vaccine AD-HPV16/18/58 mE6E7 is similar to that of wild-type adenovirus and has the ability to infect target cells, and the virus cannot replicate after entering the target cells. Therefore, it is safe as a vaccine vector, and the viral vector itself can be used as an adjuvant, so there is no need to design and add an immune adjuvant.

To evaluate the antitumor effect of the AD-HPV16/18/58 mE6E7 adenovirus vector vaccine, we need an animal model that can efficiently and stably express the HPV16, 18, 58 E6 and/or E7 genes, so C57BL/6 female mice were subcutaneously inoculated with TC-1, U14/LV-NC, U14/LV-HPV18E6E7 and U14/LV-HPV58E6E7 cell lines. RT-PCR and Western blots show that the TC-1 cells, U14/LV-HPV18 E6E7 cells and the U14/LV-HPV58 E6E7 cells can stably express the HPV16 E6, HPV16 E7, HPV18 E6E7 fusion gene and the HPV58 E6E7 fusion gene in vitro and in vivo, respectively. In the animal study of the immune effect of the AD-HPV16/18/58 mE6E7 adenovirus vector vaccine against HPV16, 18 and 58 type tumors, the results of tumor morphology showed that compared with the AD-NC empty group and PBS blank control group, the vaccine group in each cell transplantation tumor group had a later tumor formation time, a longer incubation period, and slower tumor growth; tumor growth was inhibited, and the survival time of the mice in the vaccine group was significantly prolonged. The differences were statistically significant (*P* < 0.05). The vaccine can induce immunized mice in the HPV16(+), HPV18(+), and HPV58(+) groups to produce strong humoral immunity, and the antibody titer can reach up to 1:25,600. Compared with those of the AD-NC empty group and the PBS blank control group, the antibody titers of the vaccine group of the three types of tumor-bearing mice were significantly increased, and the differences were statistically significant (*P* < 0.05). In addition, in the ELISPOT experiment of cellular immunity, the number of spots of activated IFN-γ-secreting effector T cells in the vaccine group was much greater than that of the AD-NC empty group and the PBS blank control group, and the differences were statistically significant (*P* < 0.05), which showed that mice can effectively produce specific T lymphocytes after vaccination to kill HPV16/18/58 tumor cells and protect mice from HPV16/18/58 tumor cells attacked. Then, in the specific CTL experiment, we found that the spleen lymphocytes of the AD-HPV16/18/58 mE6E7 vaccine group in the three types if tumor-bearing mice produced CTLs killing TC-1, U14/LV-HPV18 E6E7 and U14/LV-HPV58 E6E7 cells, and their highest immune protection rates were 30.160%, 30.890% and 33.050%, respectively. The killing response of the vaccine group was significantly higher than that of the AD-NC empty group and the PBS blank control group, and the differences were statistically significant (*P* < 0.05). It shows that the vaccine can activate the immune system in mice by active immunization, which can enhance mice to produce specific CTLs against E6 and E7 antigens, so as to kill HPV16/18/58 tumor cells, but have no killing effect on normal tissues, and the vaccine can inhibit tumor growth, prolong the survival time of mice, and has immune protective effect. Studies have shown that cellular immunity is more important than humoral immunity in the process of anti-HPV infection. T lymphocytes are the most critical cell subsets in tumor immunity. There are two subsets: CD4^+^ T cells and CD8^+^ T cells. CD4^+^ belongs to helper/inducible T cells, which can be divided into Th1 and Th2 subsets. CD8^+^ belongs to killer/suppressor T cells, which can kill the target cells that produce special antigen reactions, and it is the most important effector cells in anti-tumor immunity. Our results show that, the ratio of CD8^+^ T lymphocytes of vaccine group mice were significantly higher than that in the PBS and AD-NC group (*P* < 0.05), and the ratio of CD4^+^/CD8^+^ were significantly lower than those in the PBS and AD-NC group (*P* < 0.05). CD8^+^ T lymphocytes of the vaccine group in the three tumor-bearing groups were significantly higher than those of the PBS group, indicating that the AD-HPV16/18/58 mE6E7 vaccine can significantly activate the immune effect of CD8^+^ T lymphocytes in mice and inhibit CD4^+^ T lymphocytes, promote the development and activation of tumor effector cells, enhance the antitumor CTL effect, and enhance the body's antitumor ability. In summary, the AD-HPV16/18/58 mE6E7 therapeutic adenovirus vector vaccine can make immunized mice have a certain degree of anti-HPV type 16, 18, and 58 tumor activity and protect C57BL/6 mice from TC-1 and U14/LV-HPV18 E6E7 and U14/LV-HPV58 E6E7 cell attack and has a greater degree of immune protective effect. It can be used as a candidate multivalent vaccine for the immunotherapy of HPV type 16, 18, and 58-related cervical precancerous lesions and cervical cancer in the future. Venuti A et al. [35]. conducted research on a therapeutic vaccine based on the HPV16 E7 gene to protect mice from HPV16-related cancers. Zhao L et al. [33] constructed a recombinant vaccinia virus expressing the HPV18 E7E6 fusion protein in 2008 and tested its immunogenicity. Professor Wang He et al. [36]. carried out the construction and eukaryotic expression of the PVAX1-HPV58 mE6E7FcGB composite gene vaccine in 2013.

In addition, research on multivalent therapeutic cervical cancer vaccines is very common. Zhao Li [37] implemented the construction and immune effect research of a multivalent recombinant vaccinia virus against HPV types 16, 18, and 58 in 2008. Wick Darin A et al. [38] constructed a new broad-spectrum therapeutic HPV vaccine against E7 proteins of HPV types 16, 18, 31, 45 and 52, and the vaccine could induce E7-specific CD8^+^ T cell immunity and result in the regression of established large-scale E7-expressing TC-1 tumors. In this study, we selected the three most common HPV pathogenic subtypes HPV16, 18 and 58 in China to construct a new composite therapeutic vaccines, which has certain advantages in immune effect, and can protect C57BL/6 mice from the attack of HPV16 (+), HPV18 (+) and HPV58 (+) cells to a great extent. They can be used as a candidate multivalent vaccine for immunotherapy of HPV16, 18 and 58 related cervical precancerous lesions and cervical cancer in the future.

There are some shortcomings in the research on the immune effect of this new vaccine. For example, the prolongation of the survival period of immunized mice is not very obvious, and the highest value of the immune protection rate of the specific CTL response of the immunized mice was not optimal. Analyzing the above problems, we speculate that there are several reasons: First, is the vaccine delivered to the inside and outside of the host cell efficiently and stably? Second, is there a better way to modify the carrier or antigen to improve the immune effect? Relevant reports indicated that the ginsenoside monomers Rg1 and Rb1 can improve the specific immune effect of a therapeutic HPV16 recombinant vaccine in C57BL/6 mice and are expected to be candidate adjuvants [39]. Other related literature notes that a cervical cancer nanoparticle vaccine could introduce the heat shock protein (HSP)110 gene into tumor cells, which strongly improved the epitope-specific immunogenicity in vitro and in vivo, induced the proliferation of T lymphocytes and significantly enhanced the antigen-specific CTL response and its cytotoxic effect. The vaccine significantly inhibited tumor growth in mice and prolonged their survival time [40]. Research reports have also shown that vaccines and vectors encoding the immunostimulatory factors granulocyte macrophage-colony stimulating factor (GM-CSF), IL-2, and IL-12 can coimmunize mice and enhance the activation of T cells and significantly increase the intensity of immune responses of specific cytotoxic T cells [41]. According to the two key issues of vaccine construction, we can improve this vaccine in the future, modify the antigen or carrier, and improve the vaccination route, vaccination volume or immunization interval to improve the vaccine effect and safety.

HPV vaccines have made significant progress in preclinical studies and clinical trials. Compared with HPV preventive vaccines, however, HPV therapeutic vaccines still lack clinical research results. The mechanism of therapeutic vaccines is complex, and research faces many challenges. At present, whether HPV preventive or therapeutic vaccines are used, HPV types 16 and 18 are the most common, but research on the HPV58 vaccine is still scarce. In view of the special status of HPV58 in the incidence of cervical cancer in Chinese women, it is important to prepare a multivalent therapeutic vaccine containing HPV58 suitable for the Chinese population to prevent HPV infection, treat cervical precancerous lesions and even cervical cancer and prevent its recurrence by vaccination.

## Conclusion

We successfully constructed the HPV16/18/58 trivalent therapeutic adenovirus vaccine AD-HPV16/18/58 mE6E7. The AD-HPV16/18/58 mE6E7 adenovirus vaccine can protect immunized mice to a certain extent from TC-1, U14/LV-HPV18 E6E7 and U14/LV-HPV58 E6E7 cells, which contain HPV16, 18 and 58 E6 and/or E7 genes, respectively. At present, the HPV vaccines on the market are preventive, which are ineffective for patients with cervical lesions or even cervical cancer who have been infected with HPV. Besides HPV16 and HPV18, HPV58 is also the dominant HPV type for Chinese women. Therefore, there is an urgent need to develop a therapeutic vaccine containing HPV16/18/58 type to benefit Chinese patients with squamous intraepithelial lesion and cervical cancer patients suffering from high-risk HPV.

## Figures and Tables

**Fig. 6:**
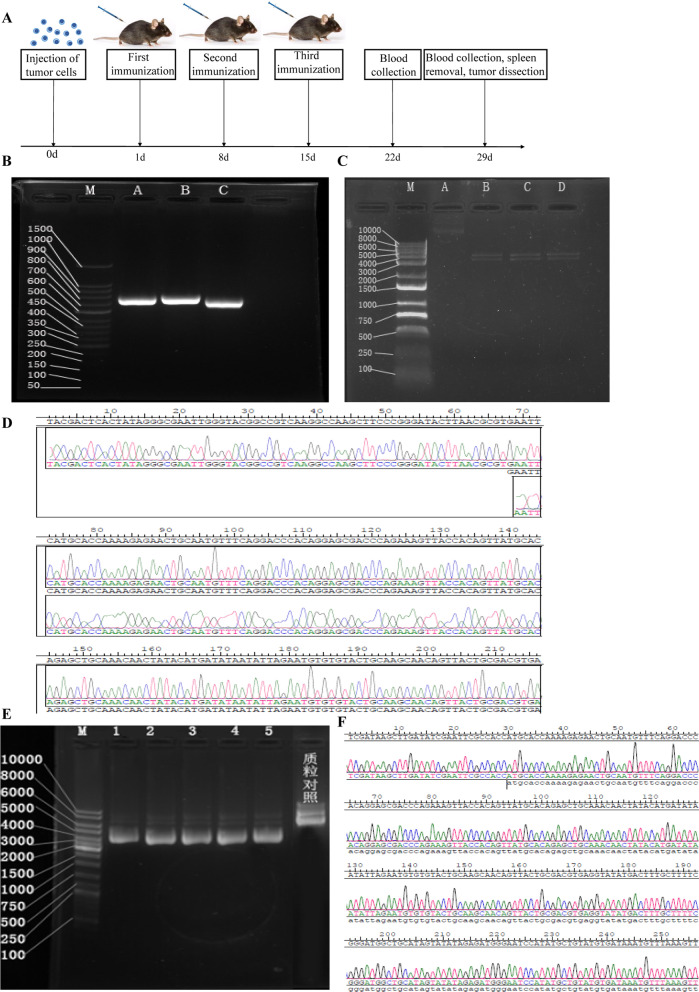
Specific immune procedures of the treatment model, the full gene of the pGH/HPV16/18/58 mE6E7 plasmid after point mutation, and verification of the recombinant adenovirus shuttle plasmid ADV4-HPV16/18/58 mE6E7. **A** Specific immune procedures of the treatment model. **B** the PCR results of the HPV16, 18, and 58 mE6E7 gene fragments, M: DNA marker (50 bp), A: HPV16 mE6E7 fusion gene (768 bp), B: HPV18 mE6E7 fusion gene (789 bp), C: HPV58 mE6E7 fusion gene (741 bp). **C** The restriction results of the HPV16/18/58 mE6E7 fusion gene fragments, and the sequencing results of the plasmid pGH-HPV16/18/58 mE6E7. M: DNA marker (5000 bp), A: plasmid pGH-HPV16/18/58 mE6E7, B, C, D: plasmid pGH-HPV16/18/58 mE6E7 after digestion. **D** Sequencing results of the plasmid pGH-HPV16/18/58 mE6E7. **E** The results of double enzyme digestion of plasmid ADV4-HPV16/18/58 mE6E7. M: DNA markers 1, 2, 3, 4, and 5: Successfully constructed recombinant shuttle plasmid ADV4-HPV16/18/58 mE6E7 (3480 bp). **F** The sequencing results and peak spectrum of the recombinant adenovirus shuttle plasmid ADV4-HPV16/18/58 mE6E7

## Data Availability

The datasets used and/or analyzed during the current study are available from the corresponding author on reasonable request.

## Appendix

See Fig. 6.

## Abbreviations

HPV: Human papillomaviruses
AD: Adenovirus-negative control
NC: Negative control
CTL: Cytotoxic T lymphocyte
RT-PCR: Reverse transcription-polymerase chain reaction
PBS: Phosphate buffered saline
VLP: Virus like particle
HLA: Human leukocyte antigen
CPE: Cytopathic effect
MOI: Multiple of infection
PFU: Plaque forming unit
GAPDH: Glyceraldehyde-3-phosphate dehydrogenase
LV: Lentivirus
ELISA: Enzyme-linked immunosorbent assay
ELISPOT: Enzyme-linked immunospot
OD: Optical density
PMA: Phorbol-12-myristate-13-acetate
LDH: Lactate dehydrogenase
SPSS: Statistical product and service solutions
RCA: Replication-deficient adenovirus
HSP: Heat shock protein
GM-CSF: Granulocyte macrophage-colony stimulating factor
